# Supplementary material for: Model-guided metabolic rewiring to bypass pyruvate oxidation for pyruvate derivative synthesis by minimizing carbon loss
Source: mSystems. 2024 Feb 5;9(3):e00839-23. doi: 10.1128/msystems.00839-23 (PMC10949502; doi:10.1128/msystems.00839-23)
Supplement: Supplemental Material — Fig. S1-S12. [file msystems.00839-23-s0001.docx]

**Supplemental Material**

**Model-guided metabolic rewiring to bypass pyruvate oxidation for pyruvate derivative synthesis by minimising carbon loss**

Yun Zhang^a*^, Xueliang Wang^a,b^, Christianah Odesanmi^a,b^, Qitiao Hu^a,b^, Dandan Li^a^,

Yuan Tang^a,b^, Zhe Liu^a,b^, Jie Mi^a,b^, Shuwen Liu^a^, Tingyi Wen^a,c*^

^a^State Key Laboratory of Microbial Resources, Institute of Microbiology, Chinese Academy of Sciences, Beijing, 100101, China

^b^University of Chinese Academy of Sciences, Beijing 100049, China

^c^Savaid Medical School, University of Chinese Academy of Sciences, Beijing 100049, China

Yun Zhang and Xueliang Wang contributed equally to this work.

*Correspondence: [zhangyun@im.ac.cn](mailto:zhangyun@im.ac.cn); wenty@im.ac.cn

**This file includes:**

Supplemental Figures S1 to S12


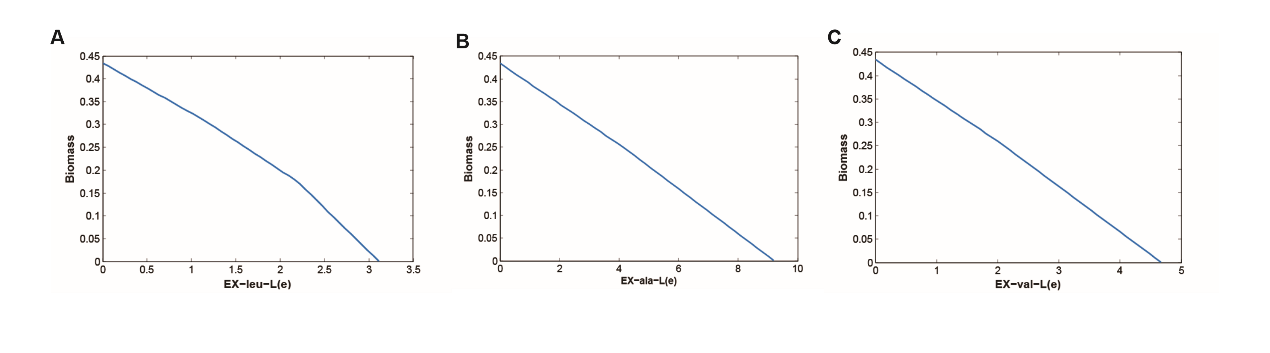


**FIG S1** The relationship between the biomass and l-leucine (A), l-alanine (B) and l-valine (C) production predicted by robustness analysis using *i*CW773 model.


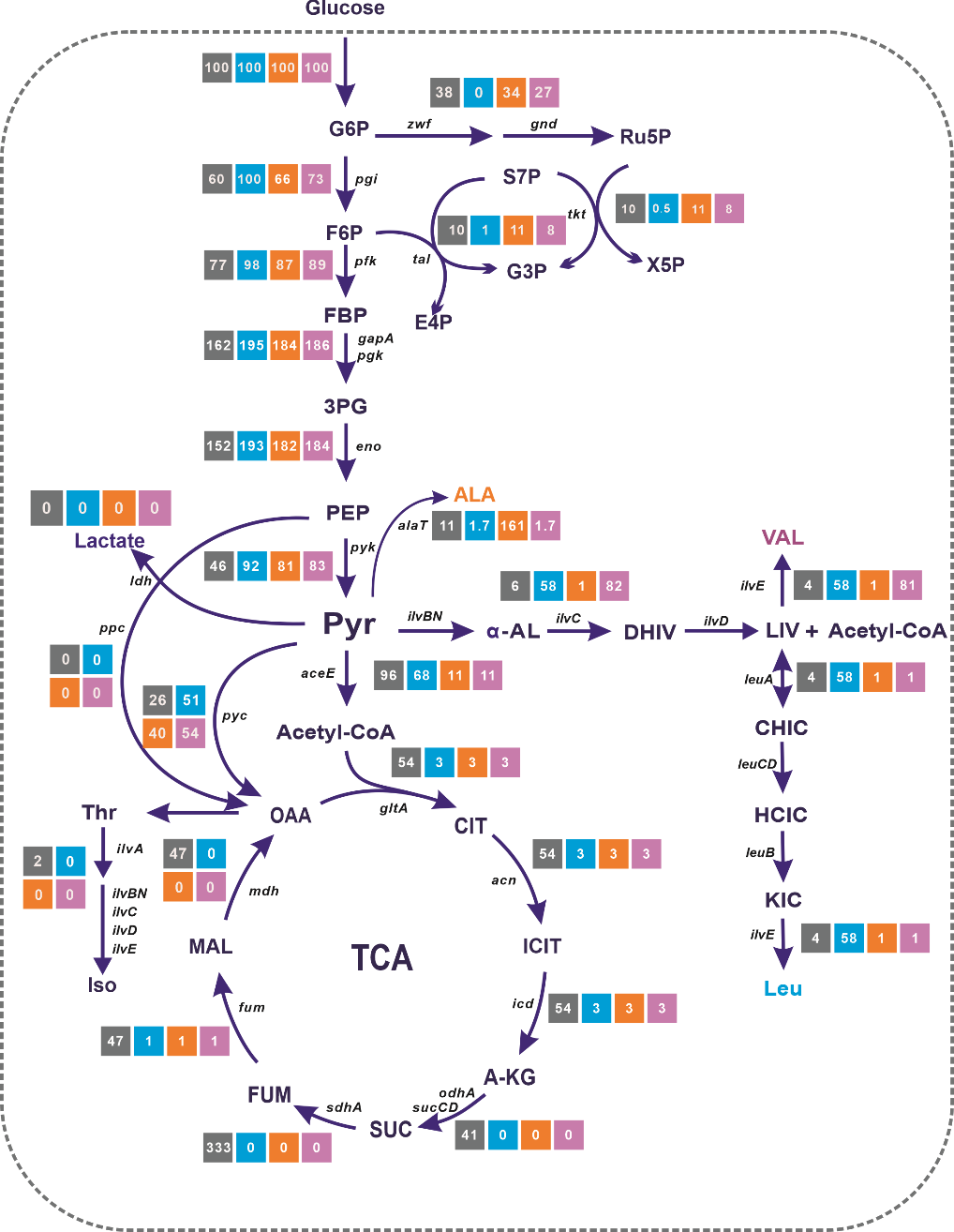


**FIG S2 *In silico* simulation of metabolic flux distribution for producing pyruvate-derived l-leucine, l-alanine, and l-valine from glucose.** Glucose uptake rate was set at 4.67 mmol/gCDW/h. The intracellular metabolic flux in WT strain was calculated using the biomass as the objective function. The lowest biomass was restricted to 20% of theoretical maximum to calculate the metabolic flux distribution in l-leucine, l-alanine, and l-valine producer using the corresponding export as the objective function. The numbers in squares represent the respective flux to the glucose uptake rate. Grey squares represent the relative metabolic fluxes for maximum biomass formation in the WT strain. Blue squares represent the relative metabolic fluxes in l-leucine producer. Orange squares represent the relative metabolic fluxes in l-alanine producer. Purple squares represent the relative metabolic fluxes in l-valine producer.


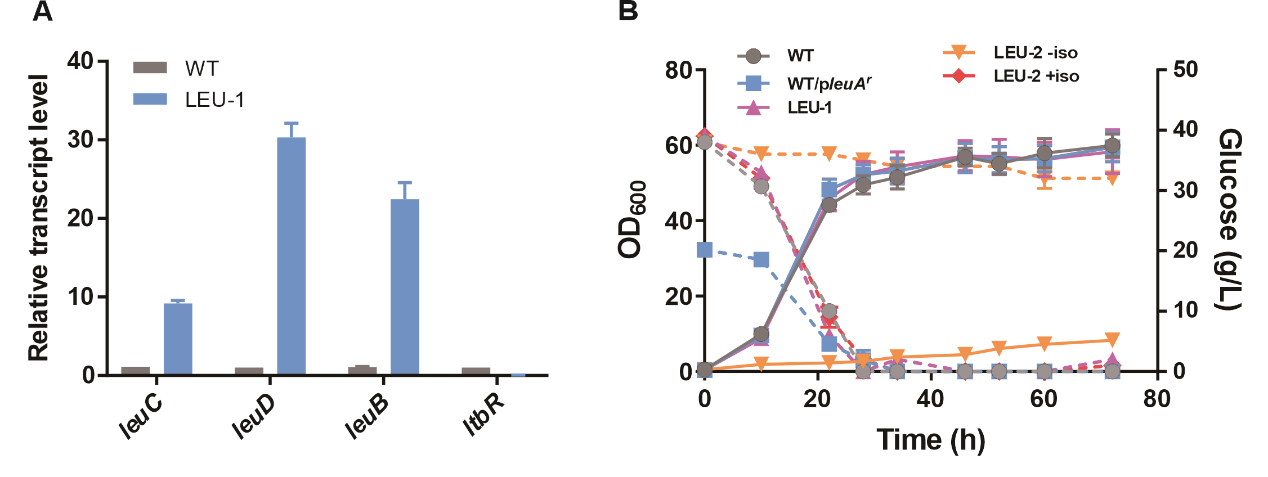


**FIG S3 The characteristics of LUE-1 and LEU-2 strains.** (A) Relative transcript levels of genes responsible for leucine biosynthesis at the exponential growth phase. (B) The growth and glucose consumption of WT, LEU-1 and LEU-2 strains in the shake flask cultivation. Isoleucine was supplemented to maintain the growth of LEU-2 strain. Data shown are mean values from three biological replicates and the standard deviations are presented.


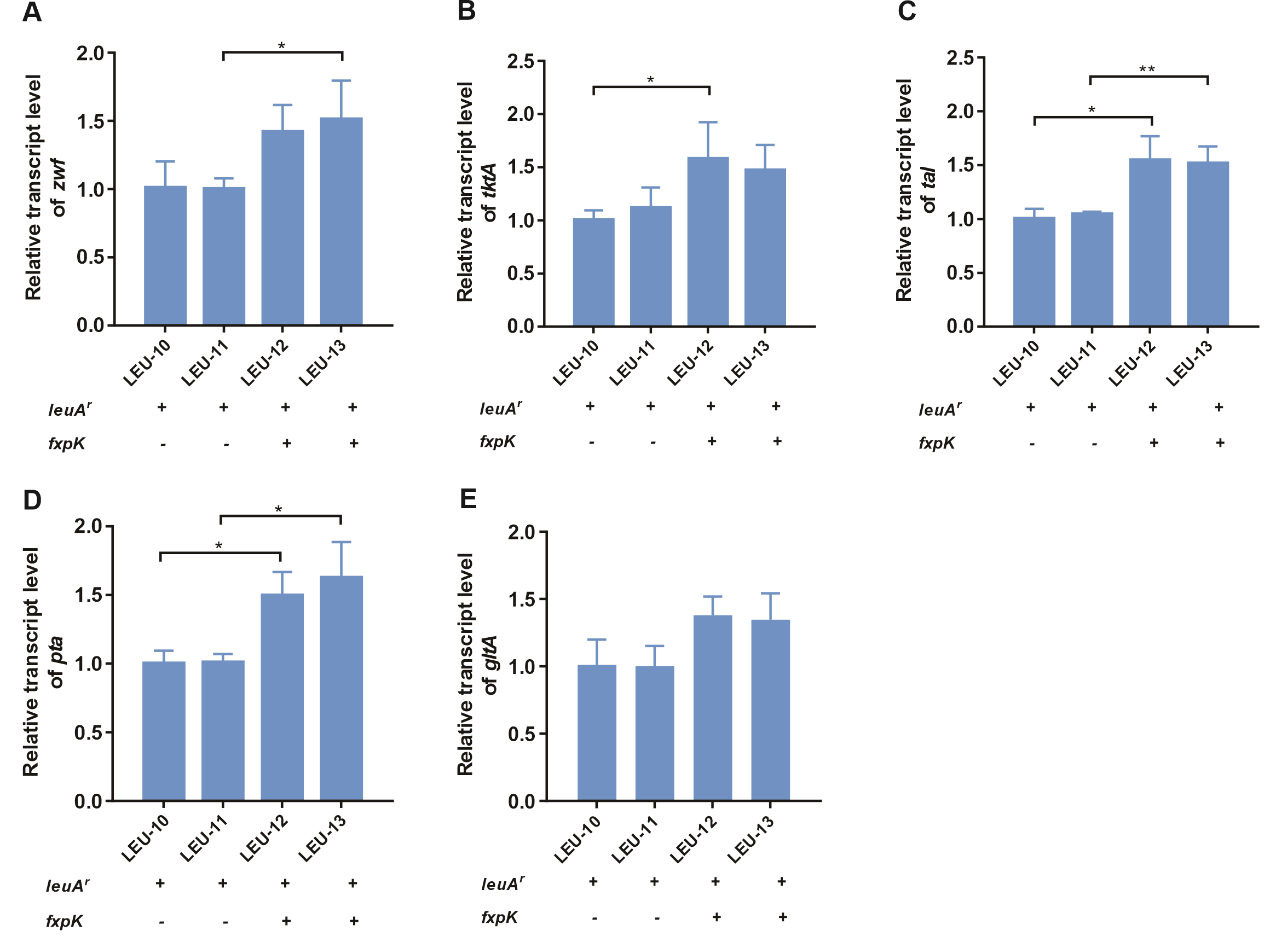


**FIG S4 RT-PCR analysis of genes in the engineered strains.** Relative transcript levels of *zwf* (A), *tkt* (B), *tal* (C), *pta* (D) and *gltA* (E) genes involved in the engineered LEU-10, LEU-11, LEU-12 and LEU-13 strains at the exponential growth phase. Data shown are mean values from three biological replicates and the standard deviations are presented. Significant differences were determined using Student’s *t* test (**P* < 0.05, ***P* < 0.01).

**
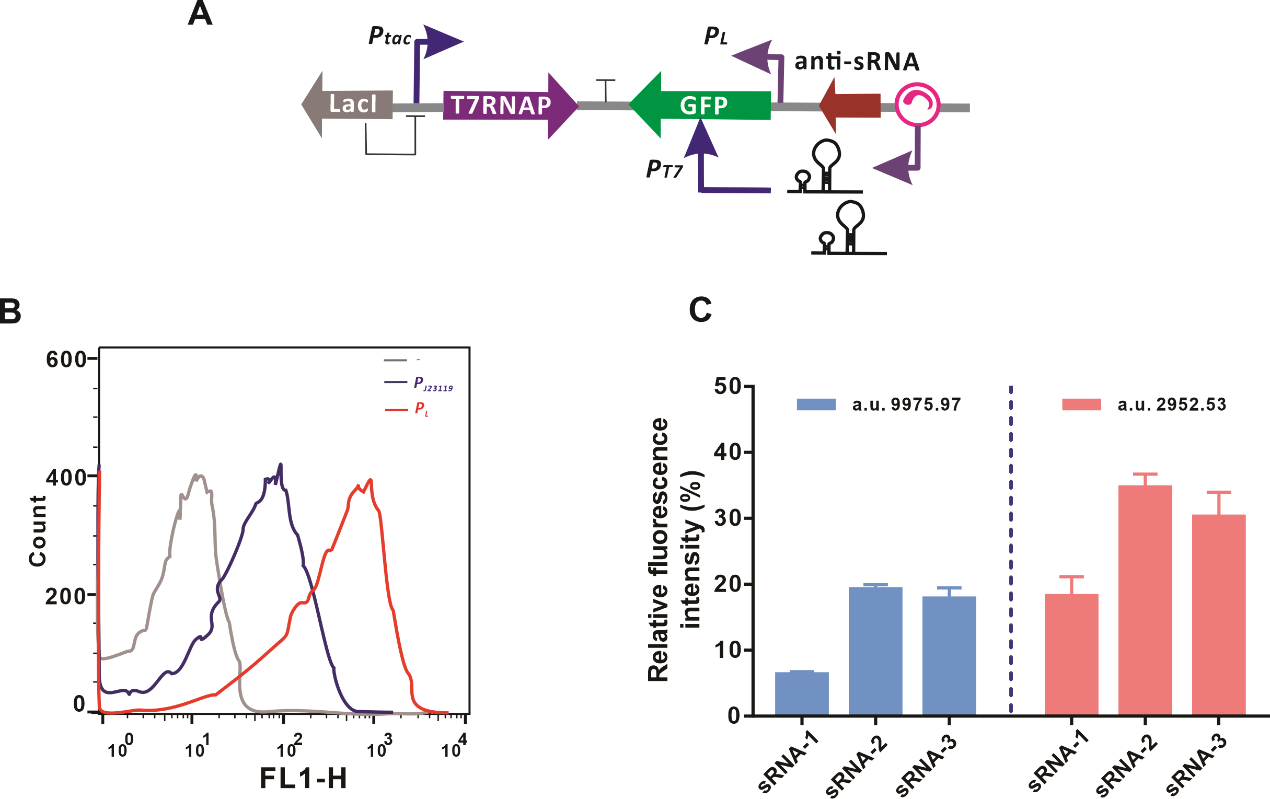
**

**FIG S5 The repression efficiency of T7 RNP-mediated sRNA on the expression of GFP under the strong *P_L_* control in *E. coli.*** (A) Scheme of T7RNAP-mediated sRNA to knockdown *P_L_*-mediated *gfp* expression. (B) Flow cytometry analysis of GFP expression under *P_J23119_* and *P_L_* controls. Histograms showing the numbers of cells and the fluorescence intensity of GFP. (C) The relative fluorescence intensity in response to three sRNAs under the control of different strength RBS-mediated T7 RNAP.


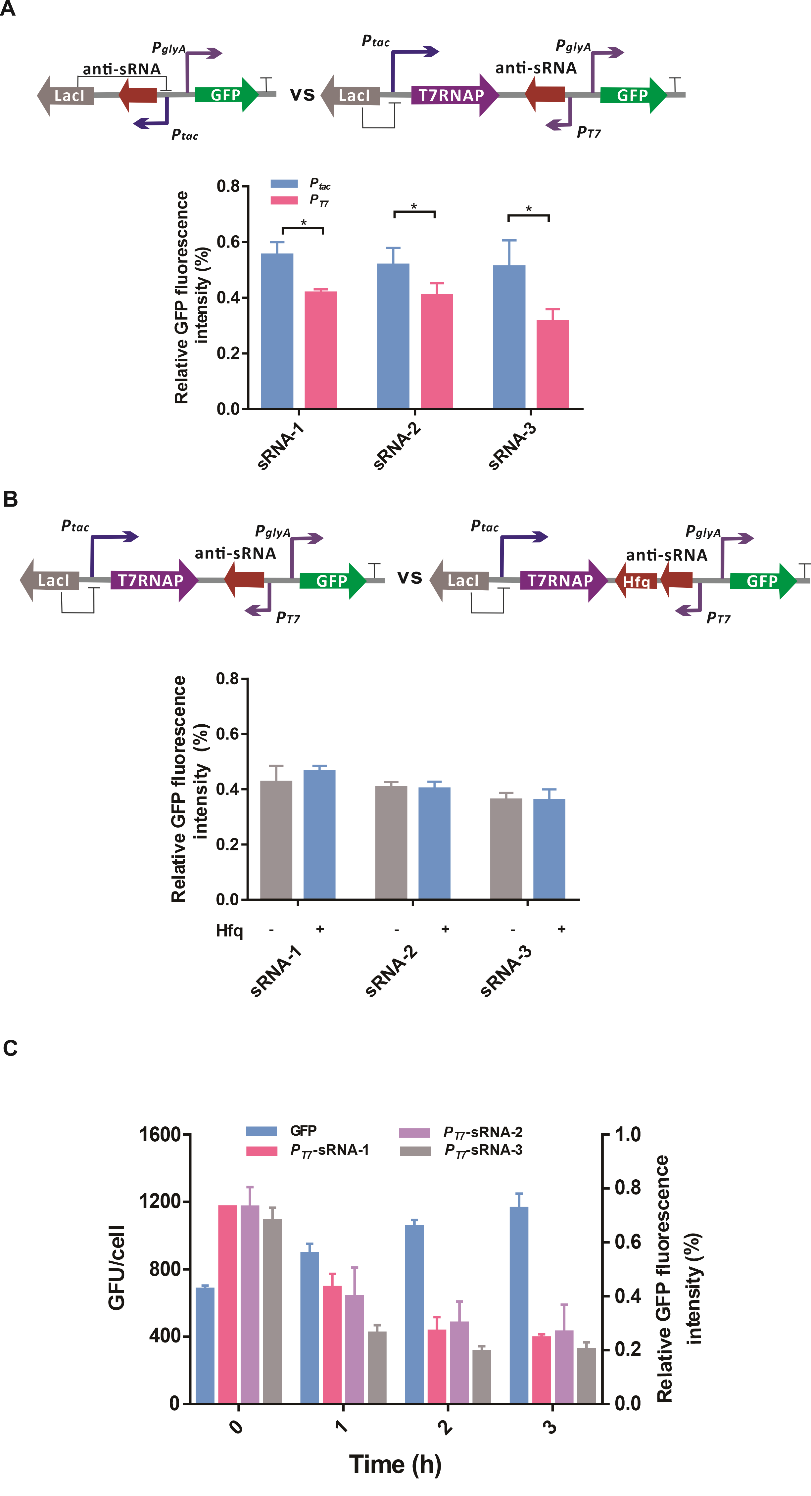


**FIG S6 The repression efficiency of T7 RNAP-mediated sRNA on the expression of *P_glyA_*-mediated GFP in *C. glutamicum.*** (A) The relative fluorescence intensity in response to the sRNA mediated by *P_tac_* and *P_T7_* promoters in *C. glutamicum*. (B) The relative fluorescence intensity of *P _T7_*-mediated sRNA on the expression of GFP in the presence and absence of Hfq in *C. glutamicum.* (C) The relative fluorescence intensity in response to the T7 RNAP-mediated sRNA in response to the inducible times in *C. glutamicum*. Data shown are mean values from three biological replicates and the standard deviations are presented.

**
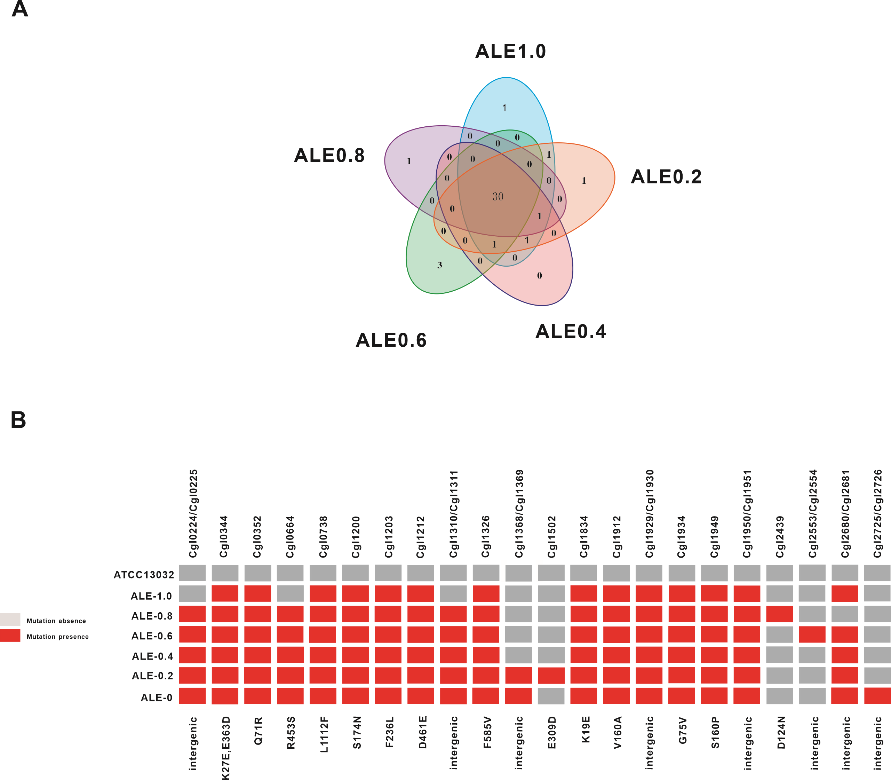
**

**FIG S7 Mutation analysis between the genomes of evolved and parental strains.** (A) Venn diagram displaying numbers of mutated genes in the five evolved population revealed by comparative whole genome sequencing. (B) Heatmap indicates frequencies of mutations in evolved population. 22 mutations cause the amino acid and regulatory sequence changes of the corresponding genes in all the evolved strains.


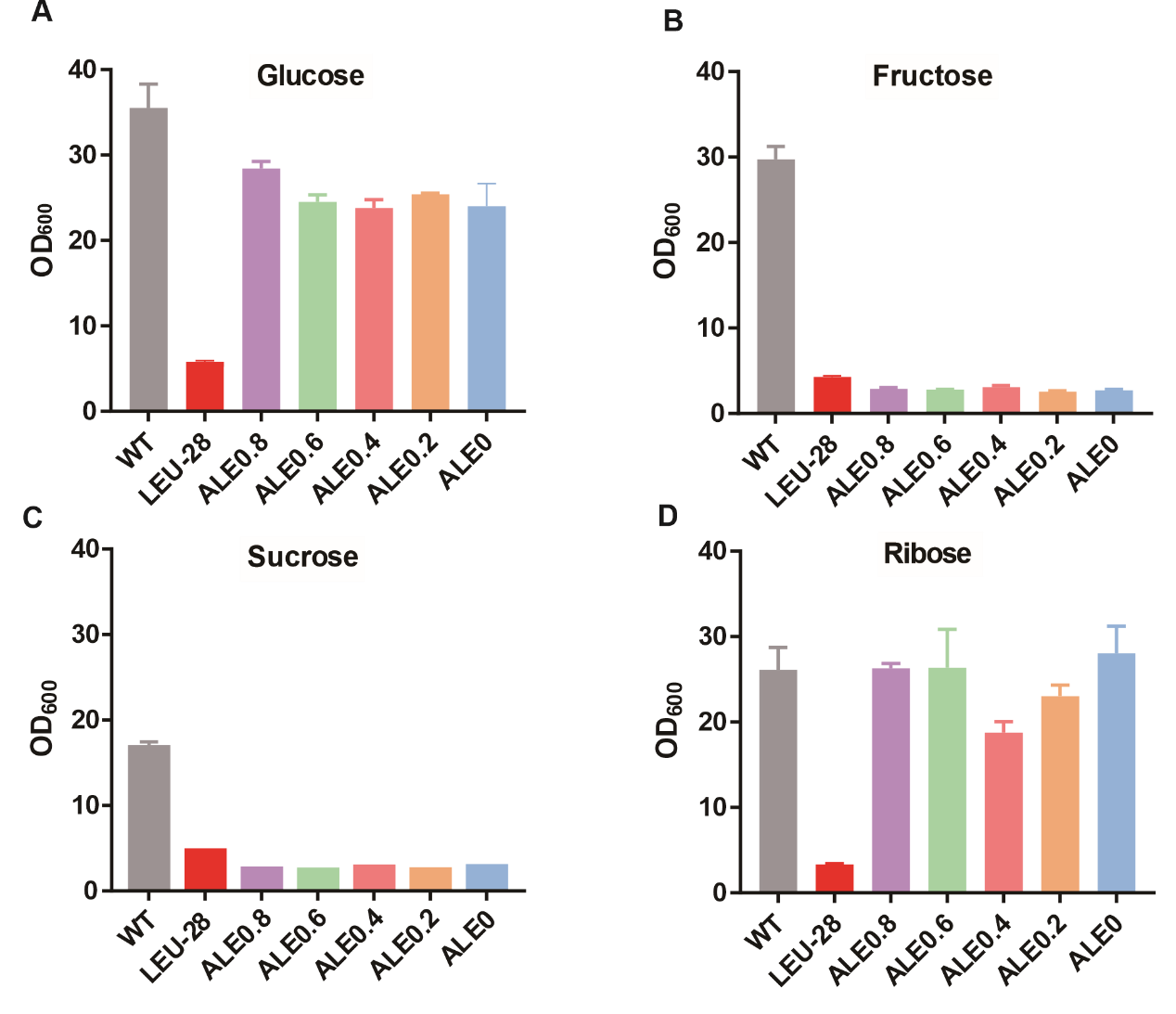


**FIG S8 The growth capacities of the evolved strains on different carbon sources. (**A) The growth of the evolved strains using glucose as a sole carbon source. (B) The growth of the evolved strains using fructose as a sole carbon source. (C) The growth of the evolved strains using sucrose as a sole carbon source. (D) The growth of the evolved strains using ribose as a sole carbon source.

**
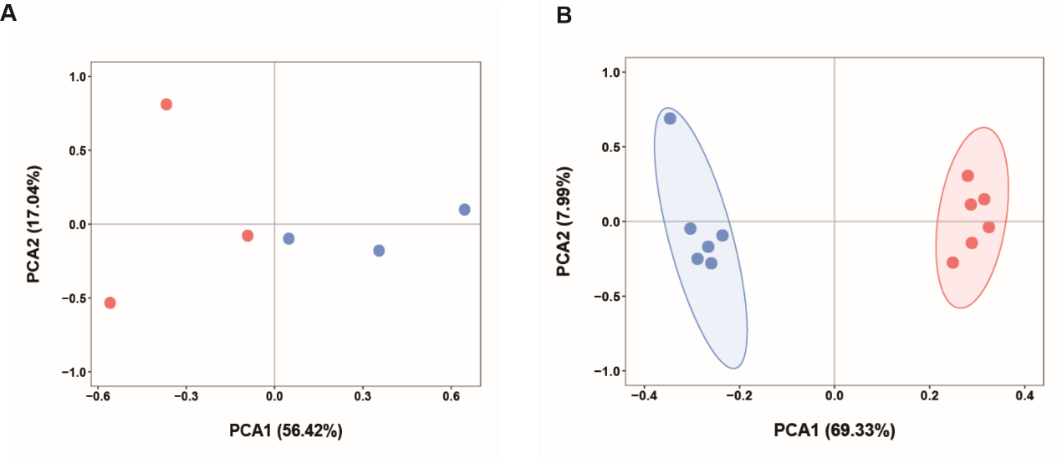
**

**FIG S9 PCA plot of transcriptomics and metabolomics.** (A) PCA plot depicting transcriptomic distances between the ALE0 and parental strains. (B) PCA plot depicting the distances of targeted metabolomics between the ALE0 and parental strains. The blue dots represent the data from the LEU-28 strain. The pink dots show the data from the ALE0 strain.


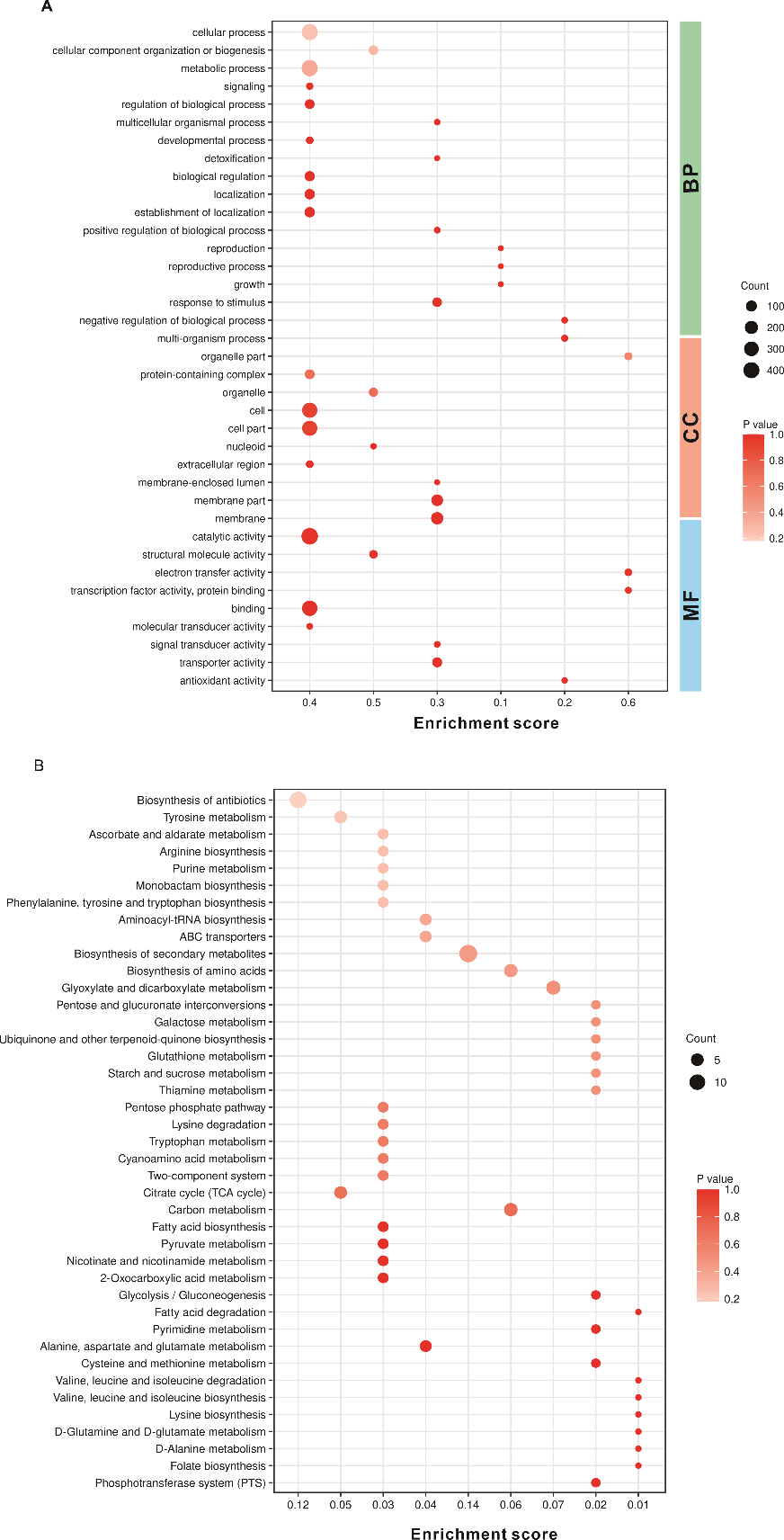


**FIG S10 Enrichment of differentially expressed genes and metabolites between the ALE0 and parental strains.** (A) Differentially expressed genes (DEGs) were annotated by Gene Ontology (GO). The Y-axis label shows GO terms. The X-axis label shows the enrichment score. (B) Differences in metabolite pools were annotated by KEGG pathways. The Y-axis label shows KEGG pathway terms. The X-axis label shows the enrichment score.


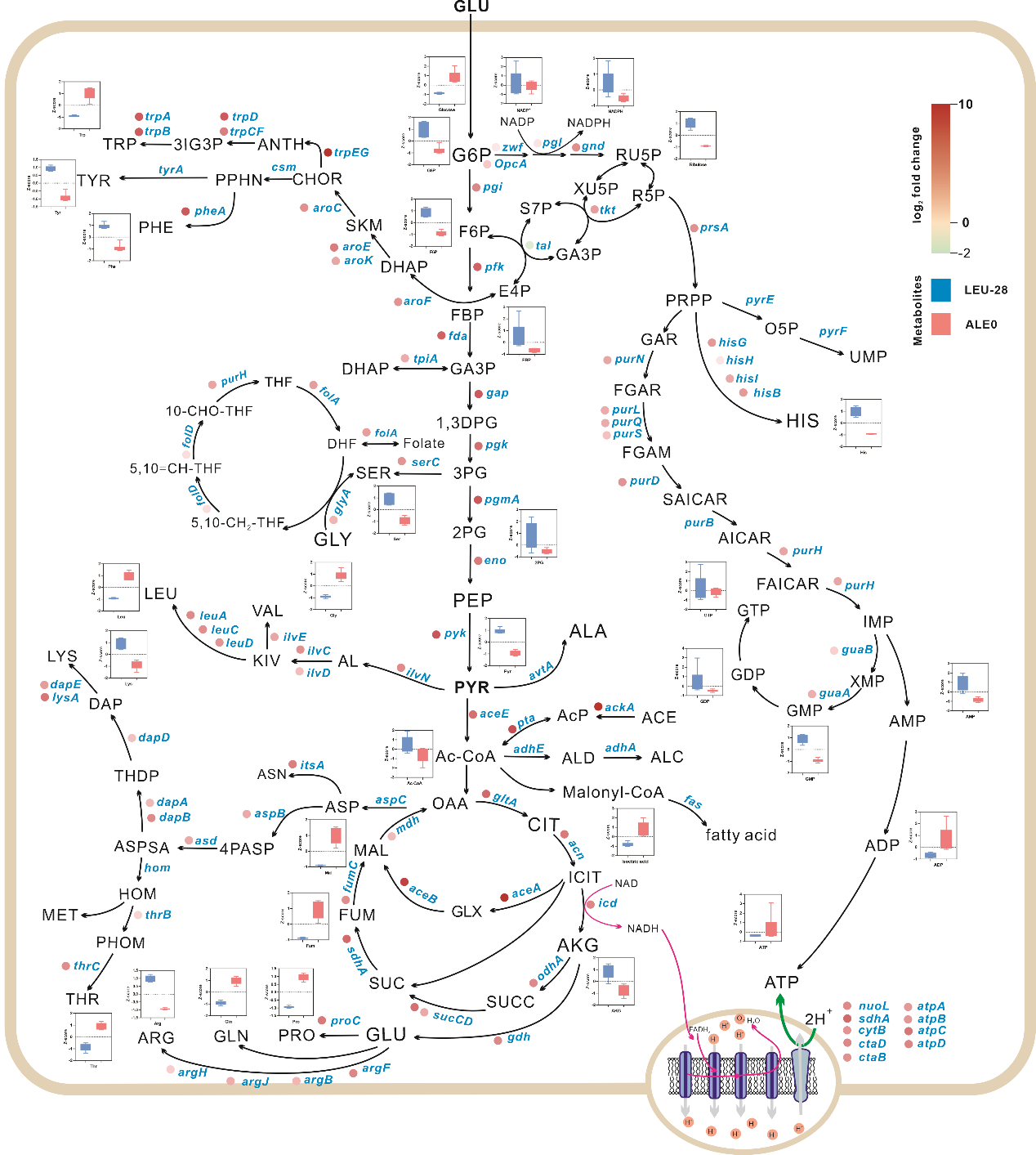


**FIG S11 Metabolic network showing transcriptomics and metabolomics analysis of relevant pathway in the LEU-28 and ALE0 strains**. Transcriptomics data are shown next to the corresponding reaction as abundance ratios between the ALE0 and the LEU-28 strains. Significant changes (1 ≥ or -1 ≤ log_2_fold in the gene transcriptional levels are depicted by colored squares. Z-score of detected metabolites are shown in column diagrams. Blue column represents the metabolite pools in LEU-28 strain, and red column represents the metabolite pools in ALE0 strain.

**
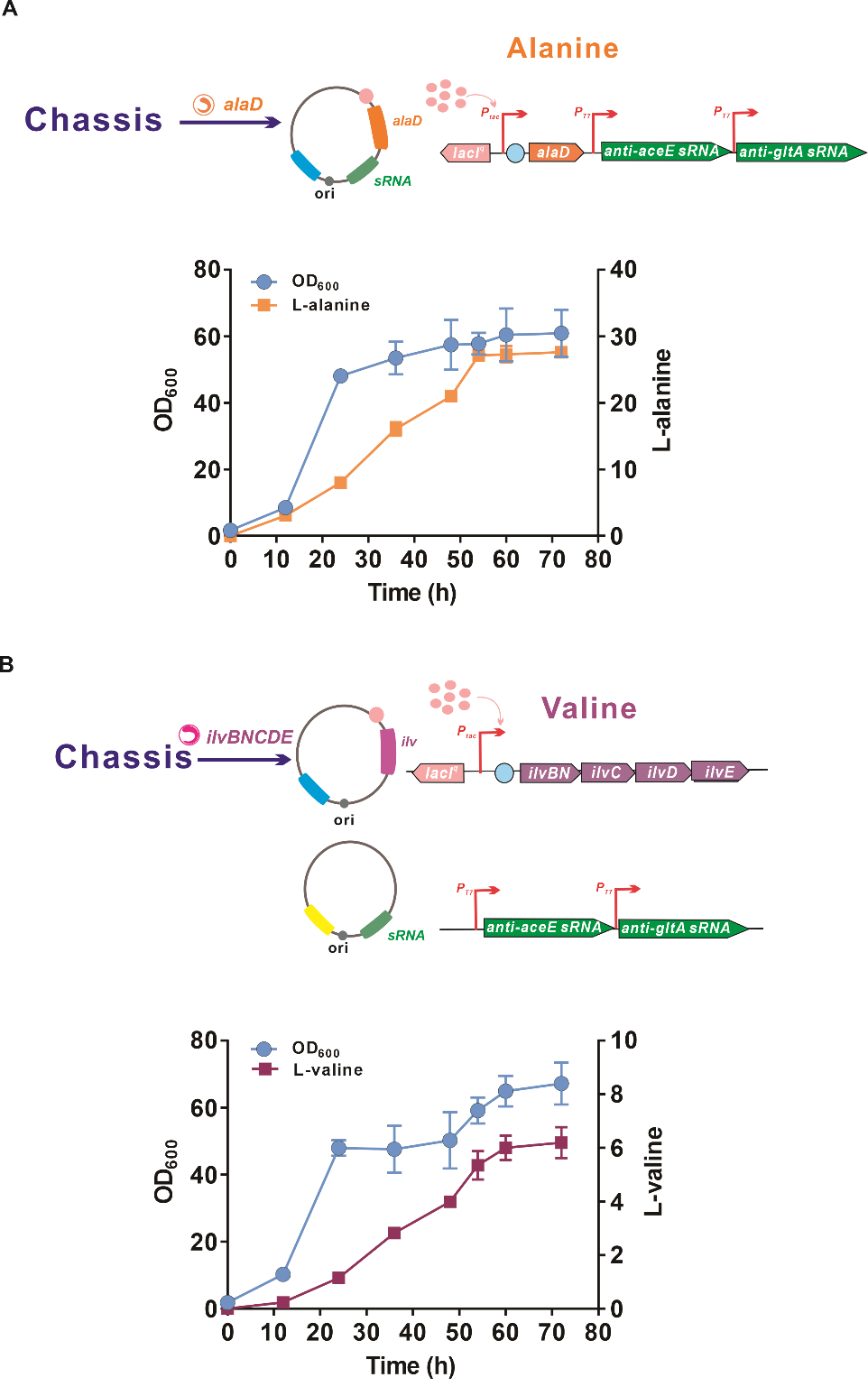
**

**FIG S12 A chassis for producing pyruvate-derived l-alanine and l-valine by a controlled switch.** (A) Time profile of cell growth and l-alanine titer in the classis strain harboring p*alaD*-*P_T7(C4)_-anti-aceE-P_T7(H9)_-anti-gltA-sRNA* in shake flask cultivation. (B) Time profile of cell growth and l-valine titer in the classis strain harboring p*ilvBNCDE* and p*P_T7(C4)_-anti-aceE-P_T7(H9)_-anti-gltA-sRNA* in shake flask cultivation.
